# Ultrahigh energy density harvested from domain-engineered relaxor ferroelectric single crystals under high strain rate loading

**DOI:** 10.1038/srep46758

**Published:** 2017-04-25

**Authors:** Sergey I. Shkuratov, Jason Baird, Vladimir G. Antipov, Evgueni F. Talantsev, Jay B. Chase, Wesley Hackenberger, Jun Luo, Hwan R. Jo, Christopher S. Lynch

**Affiliations:** 1 Loki Incorporated, Norwood, MO 65717, USA; 2 Department of Mining and Nuclear Engineering, Missouri University of Science and Technology, Rolla, MO 65409-0450, USA; 3 Pulsed Power LLC, Lubbock, TX 79416, USA; 4 Robinson Research Institute, Victoria University of Wellington, Lower Hutt 5046, New Zealand; 5 TRS Technologies Incorporated, State College, PA 16801, USA; 6 Department of Mechanical and Aerospace Engineering, University of California at Los Angeles, Los Angeles, CA 90095-1597, USA

## Abstract

Relaxor ferroelectric single crystals have triggered revolution in electromechanical systems due to their superior piezoelectric properties. Here the results are reported on experimental studies of energy harvested from (1-y-x)Pb(In_1/2_Nb_1/2_)O_3_–(y)Pb(Mg_1/3_Nb_2/3_)O_3_–(x)PbTiO_3_ (PIN-PMN-PT) crystals under high strain rate loading. Precise control of ferroelectric properties through composition, size and crystallographic orientation of domains made it possible to identify single crystals that release up to three times more electric charge density than that produced by PbZr_0.52_Ti_0.48_O_3_ (PZT 52/48) and PbZr_0.95_Ti_0.05_O_3_ (PZT 95/5) ferroelectric ceramics under identical loading conditions. The obtained results indicate that PIN-PMN-PT crystals became completely depolarized under 3.9 GPa compression. It was found that the energy density generated in the crystals during depolarization in the high voltage mode is four times higher than that for PZT 52/48 and 95/5. The obtained results promise new single crystal applications in ultrahigh-power transducers that are capable of producing hundreds kilovolt pulses and gigawatt-peak power microwave radiation.

Lead-zirconate-titanate PbZr_x_Ti_1-x_O_3_ (PZT) solid solutions discovered in the 1950s^1^ are widely used in modern ferroelectric transducers, sensors and actuators. PZT ceramics with a composition lying near a morphotropic phase boundary (MPB) between the tetragonal and rhombohedral phases exhibit high dielectric and piezoelectric properties^2^. Imperfections of crystal structure, voids and inclusions, uncontrollable crystallographic orientation of grains of PZT polycrystalline ceramics create problems for some engineering applications. Attempts to grow large-size PZT single crystals have not been successful.

Extensive studies of alternative MPB systems other than PZT led to the discovery of new relaxor ferroelectric materials, such as PIN-PMN-PT with extraordinary piezoelectric properties^3^,^4^,^5^,^6^,^7^,^8^,^9^. The successful growth of large-size relaxor single crystals led to a breakthrough in ferroelectric research^3^,^4^,^5^,^6^,^7^,^8^,^9^. The ferroelectric properties of single crystals can be tailored through domain engineering (creating domains using a specific geometric structure relative to crystallographic orientation, creating dipoles alignment that results in optimal properties).

Ferroelectric crystals have been extensively employed in the development of a new generation of sensors and transducers with improved performance^10^,^11^. There is significant interest in expanding this usage to high-power and ultrahigh-power systems, such as high intensity focused ultrasound therapy^10^, resonance based transducers^11^, and ferroelectric generators of high power microwaves^12^. High-power systems require from four to six orders of magnitude higher power than that associated with low-power transducers. It was demonstrated in ref. 13 that an increase of the stress up to a few tens of megapascals resulted in harvesting of 0.75 kJ/m^3^ energy density from PIN-PMN-PT crystals; that is significantly higher than that produced by other ferroelectrics. Studies of ferroelectric crystals under gigapascal mechanical stress provide information about ultimate energy density that can be harvested from these materials and identify possible limitations on operation in the ultrahigh-power mode.

In this paper, the results are reported on experimental studies of energy harvested from domain-engineered PIN-PMN-PT single crystals under high strain rate loading. Obtained results are directly compared to those for PZT 52/48 and PZT 95/5 ferroelectric ceramics that are widely used in modern transducers. It is shown that developed rhombohedral [111]_C_ poled PIN-PMN-PT single-domain crystals possessing highest possible remnant polarization become completely depolarized under high mechanical stress and release electric charge density significantly higher than that for PZT 52/48 and 95/5. The results indicate that the depolarization mechanism of PIN-PMN-PT crystals is distinct from those for PZT 52/48 and PZT 95/5. The PIN-PMN-PT is undergoing a ferroelectric rhombohedral-to-orthorhombic phase transition with transformation of a single-domain state into a multidomain configuration. The remarkable observation is that the energy density generated in the single crystals during depolarization in the high voltage mode exceeds 0.3 MJ/m^3^ that is four times higher than that for PZT 52/48 and 95/5. The obtained results provide the basis to the successful development of a new class of ultrahigh-power transducers and demonstrate a unique ability for precise control of ferrroelectric properties of single crystals through size and crystallographic orientation of domains to fit certain applications that is not achievable with polycrystalline ceramics.

## Methods

Lead indium niobate-lead magnesium niobate-lead titanate (25–29%)Pb(In_1/2_Nb_1/2_)O_3_-(47–35%)Pb(Mg_1/3_Nb_2/3_)O_3_-(28–36%)PbTiO_3_ single crystals were grown using the modified Bridgman technique^5^,^14^,^15^. Ternary PIN-PMN-PT single crystals at room temperature can be in either the ferroelectric rhombohedral phase or the ferroelectric tetragonal phase. These two phases are separated by a morphotropic phase boundary that can contain a monoclinic phase. When the composition is close to the MPB the ferroelectric properties of the crystals are enhanced. One of the goals of this work was to identify PIN-PMN-PT single crystals with the highest possible remnant polarization. To reach this goal the composition of the crystals, crystallographic orientation and poling conditions were varied to create domains with a specific geometric structure. The as grown PIN-PMN-PT crystals exhibit different phases along the growth direction due to the segregation of Ti. The studied specimens were selected to be in the ferroelectric rhombohedral phase and compositionally in very close proximity to the MPB, 0.27Pb(In_1/2_Nb_1/2_)O_3_–0.47Pb(Mg_1/3_Nb_2/3_)O_3_–0.3PbTiO_3_. All specimens were oriented using a real-time Laue X-ray orientation system with an accuracy of 0.5°.

The effective macroscopic symmetry associated with the domain structure is a key issue for domain engineering of single crystals. The rhombohedral phase has eight possible spontaneous polarization directions. The studied PIN-PMN-PT single crystals were poled along [001]c, [011]c and [111]c directions. For rhombohedral crystals, poling along [001]c direction induces 4*mm* macroscopic multidomain pattern symmetry, poling along [011]c direction induces *mm*2 macroscopic multidomain pattern configuration, and poling along [111]c direction induces 3*m* symmetry of the single-domain state. The most remarkable results were obtained with [111]c poled crystals. These results are described below.

The size of the PIN-PMN-PT single crystal specimens was 5.0 mm long × 5.0-mm wide × 5.0-mm thick. Vacuum sputtered Cr/Au films were deposited on the desired surfaces as electrodes. The specimens were poled along [111]c direction (3*m* symmetry) at DC electric field of 7.5 kV/cm at 50 °C. The complete set of material constants was determined by combined resonance and ultrasonic methods. Experimental details are described elsewhere^16^.

Remnant polarization of PIN-PMN-PT crystals was found to be 0.48 C/m^2^. It is significantly higher (from 30 to 90%) than that of rhombohedral and tetragonal PIN-PMN-PT crystals poled along [001]_C_ and [011]_C_ directions^16^. The higher remnant polarization of [111]_C_ poled PIN-PMN-PT crystals is attributed to the single-domain state and polarization vector being aligned normal to the electrodes in the crystals. Physical properties of PIN-PMN-PT crystals poled along [111]c direction are listed in Table 1.

Polycrystalline PZT 52/48 and PZT 95/5 ferroelectric ceramics were chosen for direct comparison with PIN-PMN-PT crystals in these studies because they are widely used for modern sensor applications and ultrahigh power transducers. Both compositions belong to PbZr_x_Ti_1-x_O_3_ binary solid solutions. PZT 52/48 and PZT 95/5 were tested under experimental conditions identical to those used for PIN-PMN-PT crystals. PZT 52/48 (trade name EC-64) ceramic specimens were obtained from ITT Corporation. PZT 95/5 ceramic specimens doped with 2% of Nb were obtained from TRS Technologies, Inc. The materials were prepared using conventional solid oxide processing (mixing oxide powders, calcining, ball milling, adding a binder, pressing, and sintering). The specimens were poled along the thickness by the manufacturers to their remnant polarization 0.29 C/m^2^ (PZT 52/48) and 0.32 C/m^2^ (PZT 95/5). The sizes and geometric shapes of the specimens affect the stress-induced behavior^16^. The specimen geometry of PZT 52/48 and PZT 95/5 was identical to that for PIN-PMN-PT crystals, 5.0 mm long × 5.0 mm wide × 5.0 mm thick, to enable a direct comparison of experimental results obtained from single crystals and ceramic materials. Properties of PZT 52/48 and 95/5 ceramics provided by the manufacturers are presented in Table 1.

**Figure 1:**
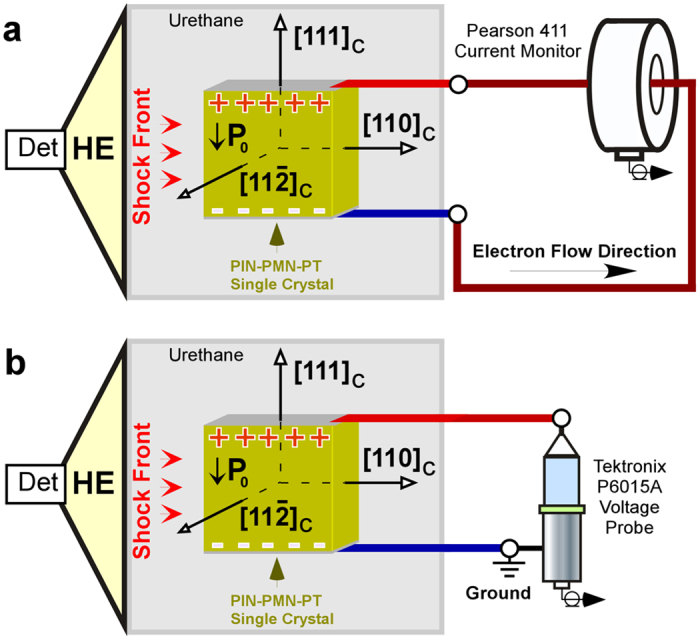
Schematics of the experimental device used for high strain rate loading studies and the measuring circuits. ***P***_0_ is the polarization vector. The polarity of the surface charge in the ferroelectric is shown by positive (+) and negative (−) signs. The loading arrangement utilizes an explosive shock compression scheme. It contains two parts, a detonation chamber and a ferroelectric specimen encapsulated within a plastic body. After initiation of the detonator, the detonation wave propagates through high explosives (HE). The detonation shock from the HE propagates through a urethane potting material and into the ferroelectric specimen. Two experimental setups were used in these investigations. (**a**) Depolarization in the short-circuit mode. The electrodes of the ferroelectric specimen were short-circuited. The compression induced a flow of electric current in the circuit that was monitored with a Pearson 411 current probe. (**b**) Depolarization in the high-voltage mode. The electrodes of the specimen were open and the compression induced high electric potential across the crystals. The voltage was monitored with a Tektronix P6015A high voltage probe.

The high strain rate loading experiments were conducted in the facilities of the Energetic Materials Research Laboratory of the Missouri University of Science and Technology, Rolla, MO. The loading arrangement and the measuring circuits used to test single crystal specimens are shown schematically in Fig. 1. This arrangement and measuring circuitry used to test PZT ceramic specimens was the same. The shock propagation direction was perpendicular to the polarization of the ferroelectric specimens (transverse compression). The single crystals were compressed along [110]_C_ direction. Additional experimental details are described elsewhere^17^,^18^,^19^.

High resolution X-ray diffraction (XRD) was performed on ferroelectric specimens using a Bragg-Brentano diffractometer (PANalytical X’Pert Pro). The XRD data were collected in a broad 2θ range (20° through 90°) using Cu *Kα* radiation (λ = 0.154060 nm). Continuous scanning mode was used and the data were recorded with a scanning step 0.017° 2θ and a scanning time of 4 seconds per step. All diffraction patterns were subjected to the fitting algorithm provided by the PANalytical X’Pert Highscore Plus software to determine the position, intensity, broadening and shape of each peak. X’Pert HighScore Plus uses the Pseudo-Voigt profile function, which is the weighted mean between a Lorentz and a Gauss function. The *d*-spacing was performed using the X’Pert Highscore FWHM single peak fitting routine.

### Stress-induced depolarization

**Figure 2:**
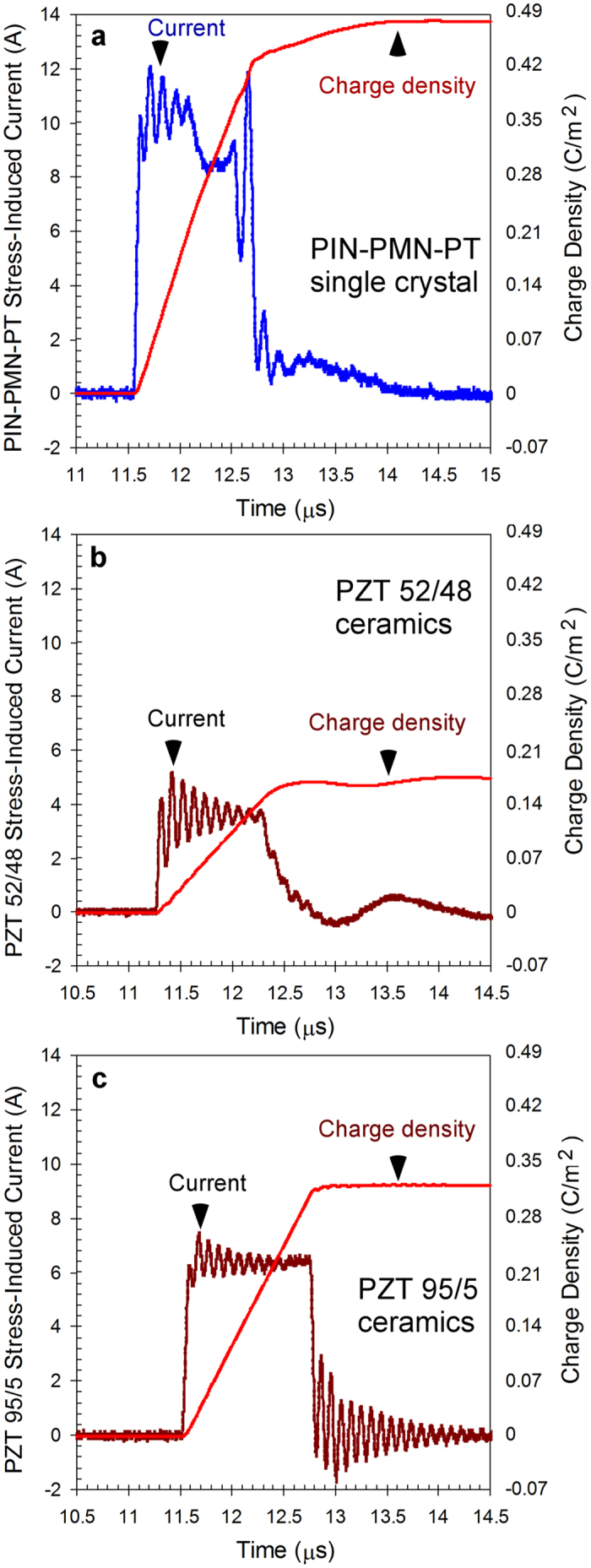
Typical waveforms of stress-induced current and dynamic of electric charge released from single crystal and ceramic specimens. (**a**) PIN-PMN-PT single crystal specimen. (**b**,**c**) PZT 52/48 and PZT 95/5 ceramic specimens, respectively. The stress-induced current waveforms are single pulses with identical direction of the current flow for PIN-PMN-PT single crystal and PZT 52/48 and PZT 95/5 ceramic specimens.

When the electrodes of the ferroelectric crystals were short-circuited (see diagram in Fig. 1a) the high strain rate loading produced current versus time profiles. The stress-induced electric charge is the time integral of the stress-induced current. Figure 2 shows typical waveforms of the current and dynamics of electric charge for the single crystal and ceramic specimens under high strain rate loading. The direction of the stress-induced current flow was identical for PIN-PMN-PT crystal and PZT 52/48 and PZT 95/5 ceramic specimens (Fig. 2).

The [110]c transverse direction for rhombohedral PIN-PMN-PT [111]c poled crystals has a negative d_31_ piezoelectric coefficient. Under the transverse shock compression the linear piezoelectric effect should increase the material polarization, but this would cause a current in the direction opposite to what was observed (Fig. 2). The direction of stress-induced current flow indicates that the charge released by PIN-PMN-PT crystals under high strain rate loading is not caused by the piezoelectric effect.

Before high strain rate loading the initial remnant polarization of the ferroelectrics is balanced by the surface charge density. When the initial remnant polarization is decreased or completely lost due to the stress-induced domain reorientation the surface charge is released at the electrodes of the specimens. The polarity of the stress-induced charge released by PIN-PMN-PT crystals and PZT 52/48 and 95/5 ceramics under stress (Fig. 2) was identical to the polarity of the surface charge that balanced the remnant polarization. These results indicate that, similar to PZT 52/48 and PZT 95/5 ceramics, the PIN-PMN-PT crystals were depolarized under high strain rate loading.

The experimental results for stress-induced charge density for PIN-PMN-PT crystals and for PZT 52/48 and PZT 95/5 ceramics are summarized in Table 1. The stress-induced charge density released by PIN-PMN-PT crystals, ω_*PIN*_ = 0.48 ± 0.02 C/m^2^ is significantly higher than that for PZT 52/48 and PZT 95/5 ceramic specimens (by a factor of 3 and 1.5, respectively).

**Figure 3:**
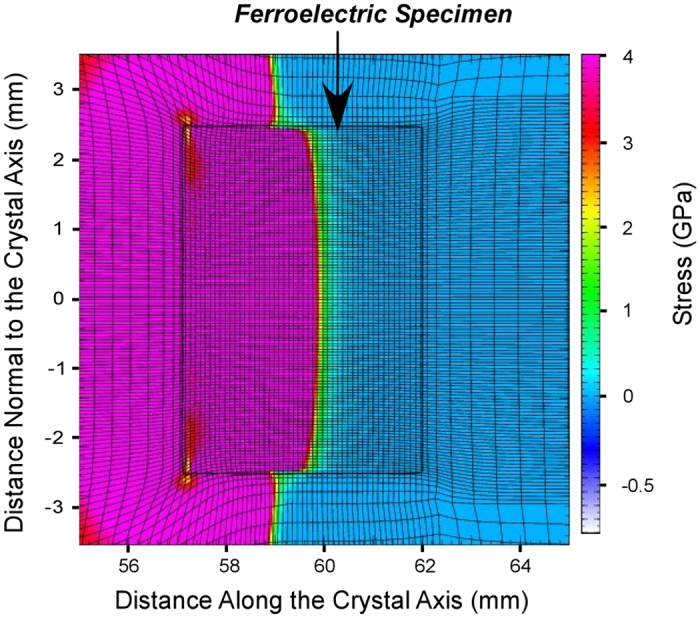
Uniaxial stress distribution in the PIN-PMN-PT single crystal specimen at 0.6 μs after compression front entered the specimen. A simulation of uniaxial stress was performed with the use of a two-dimensional Arbitrary Lagrange/Eulerian second order accurate hydrodynamics program. The simulation was run in cylindrical coordinates assuming rotational symmetry around the axis with zero material rotation.

To determine uniaxial stress distribution in the ferroelectric specimens a simulation of high strain rate loading with the use of the CALE^20^ computer code was performed. Figure 3 shows uniaxial stress distribution in the PIN-PMN-PT crystal specimen. The stress is practically uniform in the cross-section, 3.9 ± 0.1 GPa.

### Depolarization mechanism

A comparison of the stress-induced charge density released by PZT 52/48 and 95/5 ceramics with their remnant polarization (Table 1) shows a partial (52%) depolarization of PZT 52/48 and complete depolarization of PZT 95/5. These PZT 52/48 and 95/5 results are in good agreement with previous results of transverse depolarization of the two ferroelectrics^17^,^21^.

PZT 52/48 specimens were in the tetragonal ferroelectric (FE) phase. This composition lies near the MPB that separates the FE tetragonal from the FE rhombohedral structures. PZT 52/48 does not undergo a phase transition under high strain rate loading, it remains in the tetragonal phase under pressure at experimental conditions used in this work. Partial depolarization of PZT 52/48 is the result of reorientation of non-180° domains caused by release waves traveling behind the compressive front^17^.

PZT 95/5 specimens were in the rhombohedral FE phase. The composition of PZT 95/5 lies very close to a boundary between ferroelectric rhombohedral and antiferroelecric (AFE) orthorhombic phases in the phase diagram. Complete depolarization of PZT 95/5 subjected to high strain rate loading is the result of stress-induced phase transformation from FE rhombohedral to AFE orthorhombic phase^21^,^22^,^23^. This is consistent with the results of hydrostatic studies^24^. It has been demonstrated^24^ that in hydrostatically loaded PZT 95/5 the FE (rhombohedral) to AFE (orthorhombic) phase transformation occurs abruptly at a pressure of 0.32 GPa.

A comparison of stress-induced charge density and remnant polarization for PIN-PMN-PT (Table 1) shows that crystals compressed along [110]c direction were completely depolarized under high strain rate loading. Polarization can be eliminated by a phase transition to a non-polar phase, or polarization reorientation can be induced by the stress difference between the components of an applied mechanical stress that is more compressive in the polarization direction than in the transverse directions. This stress difference is the driving force for non-180° domain wall motion and polarization reorientation that result in minimization of the free energy of the system and loss of the initial remnant polarization.

Complete depolarization of PIN-PMN-PT crystals cannot be explained by domain disappearance in a non-polar cubic phase. Uniaxial pressure tends to push the crystal structure to a lower symmetry^25^,^26^. The non-polar cubic phase has higher symmetry than the uncompressed rhombohedral PIN-PMN-PT crystal. The rhombohedral-to-cubic phase transition can be reached in PIN-PMN-PT crystal by thermal heating above the Curie point or by very high uniaxial compressive stress exceeding 7 GPa^27^.

**Figure 4:**
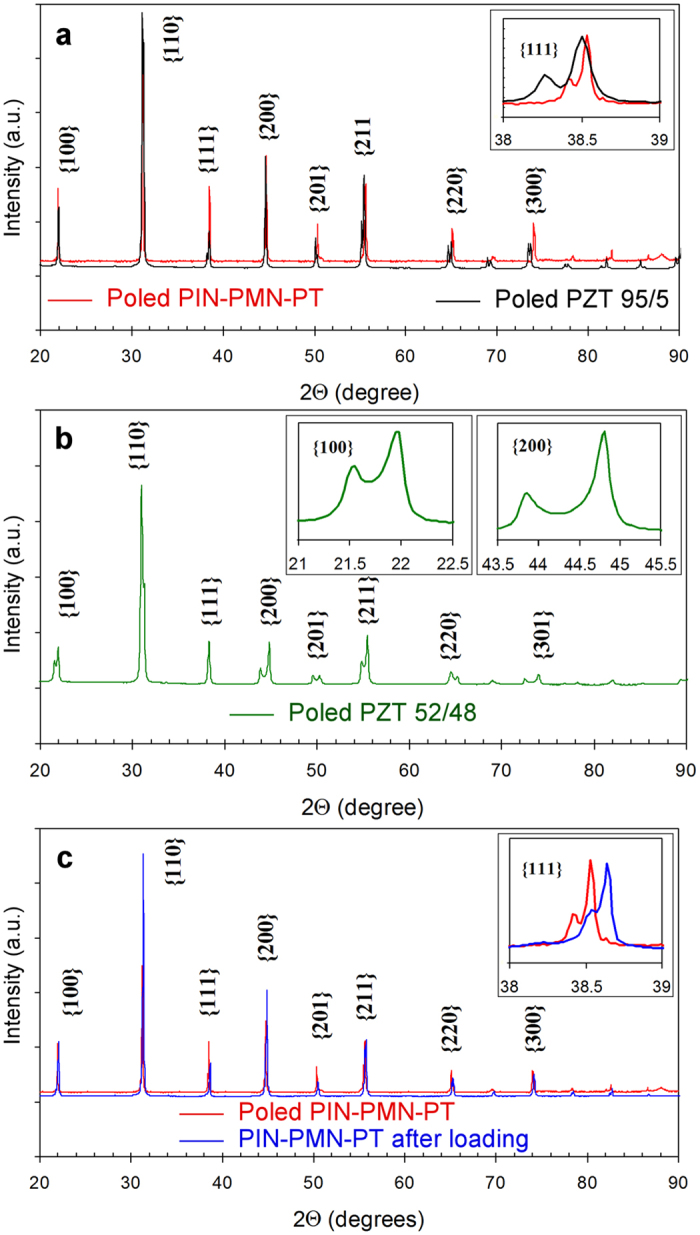
XRD patterns of single crystal and ceramic specimens. (**a**) PIN-PMN-PT and PZT 95/5 specimens in the as-received condition. (**b**) PZT 52/48 specimen in the as-received condition. (**c**) PIN-PMN-PT specimens after high strain rate loading and in the as-received condition. All specimens were crushed into powder with a mortar and pestle. All powder batches were passed through a 100-μm sieve to collect the fine particles.

X-ray diffraction was performed on ferroelectric specimens before and after high strain loading experiments. Figure 4 shows the X-ray diffraction patterns for PIN-PMN-PT, PZT 95/5, and PZT 52/48 specimens. The obtained results (Fig. 4a) indicate that XRD patterns and, correspondingly, atomic structure of PIN-PMN-PT and PZT 95/5 specimens are very similar. The peak splitting in the {111} reflections indicates a predominantly rhombohedral distortion (insert in Fig. 4a). For PZT 52/48 the peak splitting was observed in the {100} and {200} reflections in the diffraction patterns and this indicates a predominantly tetragonal distortion (inserts in Fig. 4b).

The XRD patterns of PIN-PMN-PT specimens after high strain rate loading and in the as-received condition are shown in Fig. 4c. The two XRD patterns have the identical interplanar reflections, but the peaks for the shocked specimen were shifted to the higher 2θ. This indicates that the unit cell volume is smaller in the specimen subjected to high strain loading. The {111} reflections of two specimens are compared in the insert in Fig. 4c. The double peaks were observed in both {111} reflections. This indicates that the specimen was in the rhombohedral phase after high strain rate loading. Similar result was observed for PZT 95/5 specimens. The X-ray diffraction indicates that PZT 95/5 depolarized by the high strain rate loading is in rhombohedral phase, i.e. it returns from the orthorhombic to the rhombohedral state after loading. This observation is consistent with the results of hydrostatic studies of PZT 95/5^24^. It was shown that when the pressure for hydrostatically loaded PZT 95/5 is decreased from 0.32 to 0.14 GPa, it undergoes a transformation from the orthorhombic state back to the rhombohedral state.

Based on the obtained results it appears possible that, similar to PZT 95/5, the PIN-PMN-PT is undergoing a ferroelectric rhombohedral to orthorhombic phase transition under high strain rate loading. It should be mentioned that the rhombohedral to orthorhombic phase transformation in PIN-PMN-PT crystals caused by fatigue (polarization degradation) was observed in refs 28 and 29.

The PIN-PMN-PT orthorhombic phase is a multidomain polar phase with domains having polarization aligned along [011]_C_ directions. As the results of the rhombohedral to orthorhombic phase transition the [111]_C_ single-domain state is transformed into a multidomain structure that is compressed by the unloading waves propagating behind the shock front. This leads to domain wall motion, polarization reorientation and loss of the initial remnant polarization. To minimize energy, the resulting multidomain state is reoriented to give net zero polarization.

This explanation is in agreement with results of studies of PIN-PMN-PT and PMN-PT crystals under uniaxial static stress. It was demonstrated in ref. 26 that rhombohedral [011]_C_ poled PIN-PMN-PT and PMN-PT single crystals can be driven to the orthorhombic phase by a small uniaxial compressive stress applied in [100]_C_ direction (transverse compression) or by an electric field applied in [011]_C_ direction. This phase transition was observed as a sharp decrease in the compliance, piezoelectric, and dielectric coefficients, and polarization reorientation. This phase transformation is associated with accommodation of monoclinic phase that separates the two phases in compositions near the MPB^26^.

### High voltage mode

The obtained experimental results (Table 1) indicate that the stress-induced charge density produced by PIN-PMN-PT under stress is significantly higher than that for PZT ceramics. This is the result of both a high remnant polarization of PIN-PMN-PT crystals and their ability to release all the charge under high stress.

When the electrodes of a ferroelectric specimen are open the stress-induced electric charge results in a high electric potential across the specimen. The unique ability of ferroelectric materials to generate high voltage under stress is used in a variety of modern engineering applications including ultrahigh-power transducers^12^. Compact autonomous pulsed power systems with operating voltage ranging from 100 to 500 kV are capable of producing high power microwave radiation with peak power up to gigawatt level^12^,^30^. The ability of ferroelectric crystals to produce high voltage under stress is critical for their usage in ultrahigh-power transducers.

**Figure 5:**
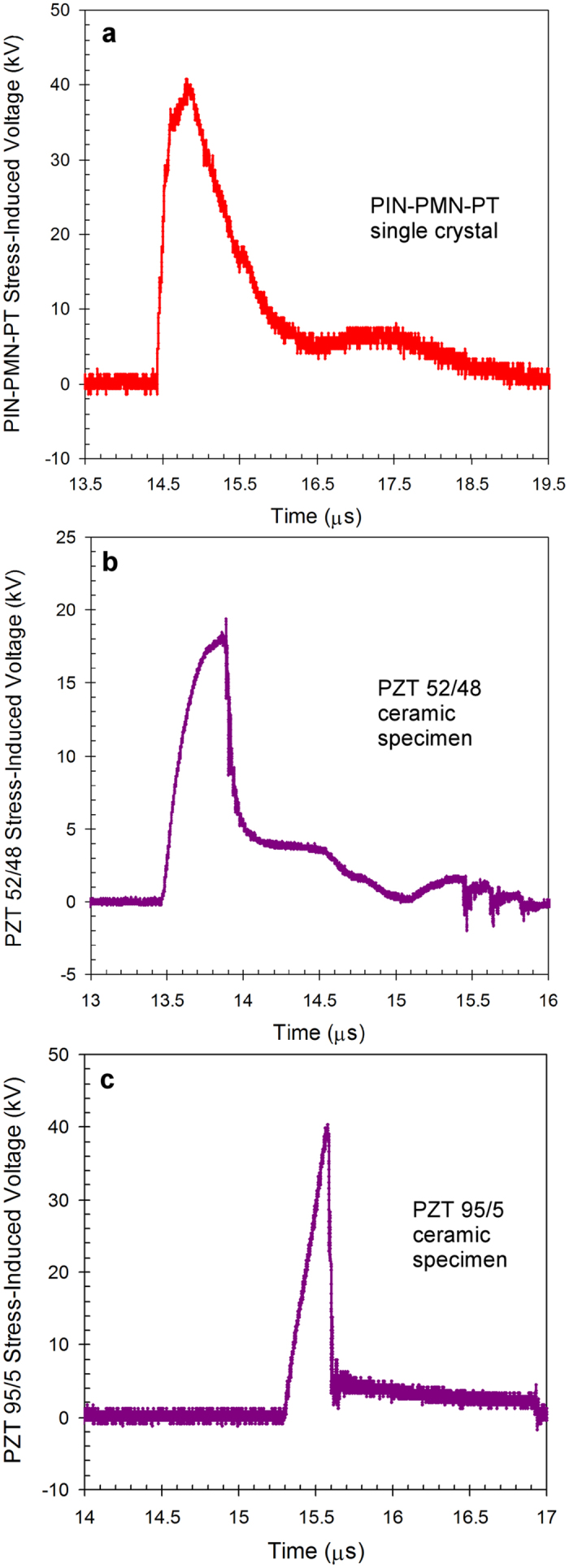
Typical waveforms of stress-induced voltage produced single crystal and ceramic specimens during depolarization in the high voltage mode. (**a**) PIN-PMN-PT single crystal specimen. (**b**,**c**) PZT 52/48 and PZT 95/5 ceramic specimens, respectively. The voltage started rising at the moment of time when the compression front entered the front face of the specimens. The voltage increased to its maximum during a few hundred of nanoseconds and then decreased due to an internal breakdown within the specimen. The experimental results indicate that the peak voltage was achieved when only a part of the ferroelectric specimens was depolarized under stress. This is typical for ferroelectric specimens of different shape and sizes.

The experiments with PIN-PMN-PT, PZT 52/48 and PZT 95/5 specimens in the high voltage depolarization mode (see diagram in Fig. 2b) were conducted. Figure 5 shows typical waveforms of the stress-induced voltage produced by the single crystal and ceramic specimens. The stress-induced voltage waveforms were single pulses with identical polarity for the three ferroelectrics. The amplitude of the voltage pulses was limited by the breakdown within the ferroelectric specimen^18^,^31^,^32^.

The PIN-PMN-PT single crystal specimen produced voltage pulses with amplitudes 41.1 kV under high strain rate loading (Fig. 5a). The corresponding electric field across the crystal was 8.22 MV/m. This is double higher voltage and electric field than those for PZT 52/48 and is practically equal to those for PZT 95/5 (Fig. 5). The PZT 52/48 and 95/5 results (Fig. 5b,c) are in good agreement with previous results obtained with the two ferroelectrics in the high voltage mode^18^,^19^,^32^. The amplitude of the voltage pulses limited by the electric strength of the ferroelectric materials (Fig. 5) can be considered as the highest possible voltage that can be produced by these single crystal and ceramic specimens under stress. The results of high voltage experiments with the three ferroelectrics are listed in Table 1.

The ferroelectric element of an ultrahigh-power ferroelectric transducer combines three stages of conventional microwave pulsed power systems in one, i.e. a prime power source, a high voltage generator, and a capacitive energy storage^12^. The energy density of a capacitive energy storage device is directly proportional to the amplitude of the voltage in the power of two and capacitance of the specimen:


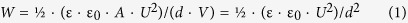


where ε is the relative dielectric permittivity (dielectric constant) of the ferroelectric, ε_0_ is the dielectric permittivity of free space (8.85 · 10^–12^ F/m), *A* is the area of the electrodes, *d* is the distance between the electrodes, *U* is the voltage across the ferroelectric specimen, *V* is the volume of the specimen. As mentioned above, complete depolarization of the entire single crystal or ceramic specimen had not occurred by the time the voltage reached the maximum (Fig. 5). To determine the upper and lower bounds of the energy density stored in each of the three ferroelectrics, the permittivity of the poled and unpoled materials (Table 1) can be used in equation (1), respectively.

Table 1 summarizes the energy density generated in the three ferroelectrics in the high voltage mode. The obtained results indicate that the energy density of PIN-PMN-PT, *W*_*PIN*_ = 0.305 ± 0.034 MJ/m^3^ is more than four times higher than that for PZT 52/48 and PZT 95/5 ceramics.

The amplitude of high voltage produced by ferroelectric specimens under high stress is increasing according to a power law with increasing thickness, *U*_g_(*d*) = γ · *d*^1–ξ^ (where *d* is the thickness of the specimen, γ is the electric breakdown field for a 1.0 mm-thick specimen, and ξ is a coefficient that is justified by the mechanism of electric breakdown)^18^,^32^,^33^. It gives the grounds to estimate the voltage and energy produced by PIN-PMN-PT: a single crystal with thickness 6 cm and total volume 70 cm^3^ is capable of producing a 400 kV microsecond pulse with total energy 15 joules. These voltage and energy levels are sufficient to provide the generation of a subnanosecond microwave pulse with hundreds megawatts peak power.

## Conclusions

The highest possible remnant polarization of (1-y-x)Pb(In_1/2_Nb_1/2_)O_3_–(y)Pb(Mg_1/3_Nb_2/3_)O_3_–(x)PbTiO_3_ single crystals was achieved by adjusting their composition and through domain engineering. The developed rhombohedral [111]_C_ poled PIN-PMN-PT single-domain crystals with remnant polarization of 0.48 C/m^2^ were subjected to high strain rate loading studies. Obtained results were directly compared to those for PbZr_0.52_Ti_0.48_O_3_ and PbZr_0.95_Ti_0.05_O_3_ ferroelectric ceramics under identical loading conditions. The polarity of the charge produced by the three ferroelectrics was opposite to the polarity of the charge generated due to the piezoelectric effect. Experimental results indicate that under 3.9 GPa stress PIN-PMN-PT crystals become completely depolarized and release electric charge density equal to its remnant polarization. The depolarization mechanism of PIN-PMN-PT crystals is different from those for PZT 52/48 and PZT 95/5. Based on the obtained results it appears possible that depolarization of the single crystals occurs due to the shock-induced rhombohedral-to-orthorhombic phase transition followed by domain wall motion and polarization reorientation caused by unloading waves propagating behind the shock front. The stress-induced electric charge provides the generation of high voltage pulses across the crystals. The important result is that the energy density generated in the crystals during depolarization in the high voltage mode exceeds 0.3 MJ/m^3^ that is four times higher than that for PZT 52/48 and PZT 95/5. Our results are a breakthrough in the research and development of ferroelectric materials for applications in ultrahigh-power transducers that generate pulses of multi-kiloampere current, hundreds of kilovolt voltage, and high power microwaves. It is direct demonstration of unique ability of the domain engineering to control precisely ferroelectric properties of single crystals to fit them certain applications, something that is not achievable with polycrystalline ceramics.

## Additional Information

**How to cite this article:** Shkuratov, S. I. *et al*. Ultrahigh energy density harvested from domain-engineered relaxor ferroelectric single crystals under high strain rate loading. *Sci. Rep.* **7**, 46758; doi: 10.1038/srep46758 (2017).

**Publisher's note:** Springer Nature remains neutral with regard to jurisdictional claims in published maps and institutional affiliations.

## Figures and Tables

**Table 1:** Physical properties of rhombohedral PIN-PMN-PT single crystals poled along [111]c direction and PZT 52/48 and PZT 95/5 ceramic specimens, and experimental results obtained with the three ferroelectrics.

Property (manufacturer data*)	PIN-PMN-PT	PZT 52/48	PZT 95/5
Density (10^3^ kg/m^3^)	8.1	7.5	7.9
Curie point (°C)	167	320	230
Dielectric constant at 1 kHz (poled)	1180	1300	295
Dielectric constant at 1 kHz (depoled)	940	1140	250
Piezoelectric constant d_33_ (10^−12^ m/V)	1800	295	68
Elastic constant s_11_^E^ (10^−12^ m^2^/N)	16.4	12.8	7.7
Remnant polarization (C/m^2^)	0.48	0.29	0.32
**Experimental result**			
Stress-induced charge density, ω (C/m^2^)	0.48 ± 0.02	0.15 ± 0.02	0.32 ± 0.02
Stress-induced voltage (kV)	40.4 ± 0.18	17.8 ± 0.16	40.1 ± 0.19
Stress-induced electric field (MV/m)	8.08 ± 0.36	3.56 ± 0.32	8.02 ± 0.38
Energy density in the high voltage mode, *W* (MJ/m^3^)	0.305 ± 0.034	0.076 ± 0.006	0.068 ± 0.004

*The tolerance of all parameters does not exceed ±5%.
